# Therapeutic Exercise Protocol During Hospitalization in Pediatric Oncohematological Patients: Randomized Clinical Trial

**DOI:** 10.1002/pri.70324

**Published:** 2026-08-18

**Authors:** Cintia Freire Carniel, Bruna Cunha de Souza, Henrique Ferreira Leite, Juliana Zangirolami‐Raimundo, Rodrigo Daminello Raimundo

**Affiliations:** ^1^ Laboratório de Delineamento de Estudos e Escrita Científica Centro Universitário FMABC Santo André São Paulo Brazil

**Keywords:** exercise, fatigue, hematologic neoplasms, muscle strength, pediatrics, quality of life, resistance training

## Abstract

**Background:**

Leukemias, lymphomas, and central nervous system tumors are among the most common pediatric cancers and may lead to motor deficits, impaired balance, reduced muscle strength, fatigue, and decreased functional capacity. Early physiotherapy during hospitalization may help prevent inactivity and support functional preservation in this population.

**Objective:**

To evaluate the effects of a therapeutic exercise program on quality of life, muscle strength, fatigue, and functional capacity in hospitalized pediatric oncohematological patients.

**Methods:**

Thirty participants aged 8–17 years with oncohematological diseases were randomized to an intervention group (IG) or a minimal active physiotherapy comparator group (CG). Assessments included the 6‐min walk test, handgrip dynamometry, the PedsQL Multidimensional Fatigue Scale, and the PedsQL Cancer Module at admission and discharge. The IG performed daily 25‐min supervised sessions including aerobic, resistance, and breathing exercises with ambulation guidance, whereas the CG received breathing exercises and ambulation guidance.

**Results:**

No significant group × time interactions were observed for total fatigue or its domains, overall quality of life or its assessed domains, handgrip strength, or six‐minute walk test distance. Time‐related changes were observed for some outcomes, but these occurred without evidence of differential change between groups and were not interpreted as effects of the structured exercise protocol. No intervention‐related adverse events requiring permanent protocol discontinuation were recorded.

**Conclusion:**

The structured in‐hospital therapeutic exercise protocol could be delivered under close clinical supervision without recorded intervention‐related adverse events requiring permanent discontinuation. However, the structured protocol did not demonstrate superiority over the minimal active physiotherapy comparator for fatigue, quality of life, muscle strength, or functional capacity. These findings should be interpreted cautiously because of the small sample size, clinical heterogeneity, variable intervention exposure, and limited intervention‐fidelity data.

**Trial registration:**

Brazilian Registry of Clinical Trials (ReBEC), RBR‐8sxnfyd

**Abbreviations**
ACT: Arm Curl Test
AF: Assent Form
ALL: Acute Lymphoblastic Leukemia
ANOVA: Analysis of Variance
BMT: Bone Marrow Transplantation
Borg: Borg Rating of Perceived Exertion Scale
CG: Comparator Group
CO: Cardiac Output
CONSORT: Consolidated Standards of Reporting Trials
CTX‐D: Cancer and Treatment Distress
DBP: Diastolic Blood Pressure
EORTC‐QLQ‐C30: European Organization for Research and Treatment of Cancer Quality of Life Questionnaire Core 30
FAB: Fullerton Advanced Balance Scale
FACT‐BMT: Functional Assessment of Cancer Therapy–Bone Marrow Transplant
HDC: High‐Dose Chemotherapy
HR: Heart Rate
HRmax: Maximum Heart Rate
HSCT: Hematopoietic Stem Cell Transplantation
HSD: Honest Significant Difference
ICF: Informed Consent Form
ICU: Intensive Care Unit
IG: Intervention Group
INCA: National Cancer Institute
IS: Immobility Syndrome
MAP: Mean Arterial Pressure
MFIS: Modified Fatigue Impact Scale
PedsQL: Pediatric Quality of Life Inventory
PSQI: Pittsburgh Sleep Quality Index
REBEC: Brazilian Registry of Clinical Trials
RR: Respiratory Rate
SBP: Systolic Blood Pressure
6MWT: Six‐Minute Walk Test
SMI: Sustained Maximal Inspiration
SPA: Standardized Phase Angle
SpO_2_
: Peripheral Oxygen Saturation
SPSS: Statistical Package for the Social Sciences
SV: Stroke Volume
TUG: Timed Up and Go
VAS: Visual Analog Scale
VCO_2_
: Carbon Dioxide Production
VE: Minute Ventilation
VO_2_
: Oxygen Consumption

## 1 Introduction

Childhood cancer results from the uncontrolled proliferation of atypical cells and affects individuals up to 19 years of age. Children aged 0–4 years are at the greatest risk of developing cancer; however, lymphomas, carcinomas, and bone tumors are more common among individuals aged 10–14 years. The hematopoietic system and supporting tissues are the most frequently affected sites although cancer may develop in any part of the body. Unlike adult cancers, in which environmental factors play an important role in tumor development, there is no consistent scientific evidence that such factors alone are responsible for the onset of childhood cancer. Therefore, childhood cancer is generally considered to originate during embryonic development (INSTITUTO NACIONAL DE CÂNCER JOSÉ ALENCAR GOMES DA SILVA 2019; Zuchetti and et al. 2018).

In some developing countries, children account for up to 50% of the population, and pediatric cancers represent between 3% and 10% of all neoplasms. In contrast, this proportion is approximately 1% in developed countries (MINISTÉRIO DA SAÚDE 2023). Overall, childhood cancers account for 1%–4% of all neoplasms in the general population, with leukemia (28%), central nervous system tumors (26%), and lymphomas (8%) being the most prevalent cancer types among individuals aged 0–19 years (American Thoracic Society 2002).

The most used chemotherapeutic agents for the treatment of hematologic cancers include cyclophosphamide, methotrexate, and vincristine, which are frequently associated with adverse neurological, cognitive, and motor effects. Children exposed to these agents may develop complications such as osteopenia, osteonecrosis, peripheral neuropathy, reduced ankle range of motion, impaired gross and fine motor skills, balance disturbances, decreased muscle strength, and fatigue. Fatigue may affect 60%–100% of patients during and after treatment (Zuchetti and et al. 2018; Tanner et al. 2017; Souza et al. 2025).

Studies have shown that children and adolescents diagnosed with cancer are considerably less active than their healthy peers, with physical inactivity increasing during hospitalization. Therefore, implementing exercise interventions during the hospital stay may be important to reduce inactivity and support functional preservation (Rustler and et al. 2017; de Souza et al. 2024; Souza et al. ^2024^).

Although the literature remains limited, some studies have investigated physiotherapeutic interventions in these patients, including stretching, strengthening, resistance exercises, balance training, gait training, and cardiorespiratory conditioning (Rustler et al. 2017). Exercise may contribute to improved quality of life, particularly by reducing fatigue levels (Su et al. 2018).

Therapeutic exercises supervised by physiotherapists can positively influence the course of the disease, playing an important role in preventing secondary complications arising from long‐term effects such as chronic fatigue or cardiorespiratory conditions. It is essential to adapt the exercises daily to the patient's clinical condition during hospitalization for oncology treatment, taking into account both the patient's current clinical status and the treatment's side effects (Götte et al. 2014).

A sedentary lifestyle, reduced mobility, prolonged bed rest, and confinement to bed contribute to a progressive decline in physical activity and may lead to the development of immobility syndrome (IS). This syndrome comprises a series of physiological alterations that affect individuals who remain seated or bedridden for extended periods, resulting in reduced functional capacity across multiple systems, including the musculoskeletal, integumentary, respiratory, cardiovascular, gastrointestinal, genitourinary, and nervous systems. It manifests through a range of signs and symptoms arising from multisystem dysfunction, primarily associated with reduced delivery of nutrients and oxygen through the cardiovascular system, whose performance is closely related to the individual's level of physical activity (Vital 2017; Abo et al. 2021).

According to Noble et al. (2012), previous studies suggest that exercise during acute cancer treatment may be feasible and safe, including during myeloablative therapy, with potential benefits for functional capacity, fatigue, and quality of life. Despite the heterogeneity of exercise programs, the principles underlying muscle overload prescription are similar across many hospitalized patients. This highlights the need for studies that assess the optimal dose, sequence, and combination of training modalities to counteract the adverse effects associated with high‐dose chemotherapy during prolonged hospitalization, as well as the challenges of conducting adequately powered studies in this population. A Cochrane review by Braan (2016) reported that exercise interventions may have beneficial effects on physical fitness, bone mineral density, flexibility, cardiorespiratory fitness, muscle strength, and quality of life.

The heterogeneity observed across studies may be attributed to differences in exercise interventions, including their mode, intensity, frequency, duration, and setting; the use of diverse outcome measures, including quantitative, qualitative, physical, and psychosocial assessments; and the methods used to evaluate intervention effects. Establishing a greater consensus regarding these elements is essential to facilitate comparisons across studies.

High‐quality evidence is still needed to develop guidelines on exercise, physical activity, and hospital‐based physiotherapy for this population. Therefore, further well‐designed trials with larger sample sizes and adequate statistical power are required. The aim of this study was to evaluate the effects of a therapeutic exercise program on quality of life, muscle strength, fatigue, and functional capacity in pediatric oncohematological patients during hospitalization.

## 2 Method

This study was reported according to the CONSORT (Consolidated Standards of Reporting Trials) guidelines.

### 2.1 Type, Location and Study Population

This randomized clinical trial was conducted in the pediatric ward of Mario Covas Hospital. The study was approved by the Research Ethics Committee (approval #5.439.594), and the protocol was registered in the Brazilian Registry of Clinical Trials (ReBEC; RBR‐8sxnfyd) on January 25, 2023. Participants were hospitalized pediatric patients with oncohematological conditions who were selected according to the eligibility criteria described below.

### 2.2 Eligibility Criteria

Participants aged between eight and 17 years, with a confirmed diagnosis of oncohematological disease, admitted to the pediatric ward of the Mário Covas State Hospital, of both sexes, were included. Participants had to be clinically stable regarding any pre‐existing respiratory, cardiac, or musculoskeletal conditions prior to the oncological diagnosis, agree to participate in the study, have their guardians sign the informed consent form (ICF), and sign the assent form (AF) themselves.

The non‐inclusion criteria were based on participants with cognitive impairments that made evaluation impossible, and on those presenting one or more of the following: body temperature above 38°C, pain rated at 5 or higher on the Visual Analog Scale (VAS), persistent diarrhea, uncontrolled bleeding, tachycardia (heart rate above 120 bpm at rest), and/or hypotension with a mean arterial pressure (MAP) below 60 mmHg. Participants with hemoglobin levels below 8.0 g/dL in the complete blood count accompanied by symptoms of decompensation, and those with platelet counts below 20,000/mm^3^ were also excluded.

### 2.3 Randomization, Allocation and Blinding

After enrollment and baseline assessment, participants were randomized to either the intervention group, which received the structured therapeutic exercise protocol, or the comparator group, which received the minimal active physiotherapy intervention described in Section 2.5. Randomization was performed using sealed opaque envelopes containing the group allocation. The envelopes were opened only after participant enrollment and baseline assessment, thereby maintaining allocation concealment before assignment.

In the study setting, hospitalized children without respiratory symptoms were not routinely prescribed physiotherapy by the attending medical team. Therefore, the comparator group should not be interpreted as receiving an established physiotherapy standard of care, usual care, or no treatment. Instead, it represented a minimal active physiotherapy intervention consisting of conventional respiratory exercises and ambulation guidance.

Due to the nature of the physiotherapy interventions, participants and treating physiotherapists could not be blinded after allocation. Participants received different exercise approaches, and the physiotherapists responsible for treatment delivery needed to know the assigned group to apply the corresponding protocol. However, outcome assessments and reassessments were performed by physiotherapists who were not involved in treatment delivery and were blinded to group allocation.

Participants were treated individually in their hospital rooms to minimize contamination between groups and reduce communication about the intervention content. Therefore, this trial used allocation concealment before assignment and blinded outcome assessment, but it was not possible to blind participants or treat physiotherapists after randomization.

**FIGURE 1:**
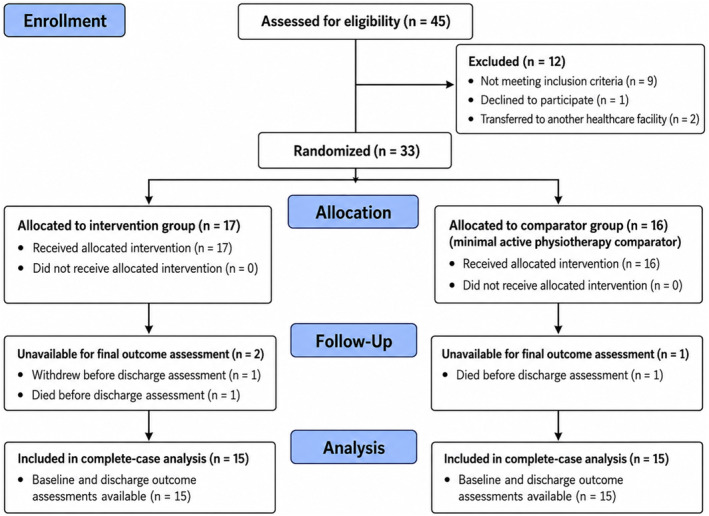
CONSORT flow diagram showing participant eligibility, exclusions, randomization, allocation, availability for the outcome assessment, and inclusion in the complete‐case analysis.

The recruitment and randomization steps are described in the CONSORT flowchart (Figure 1).

Peripheral oxygen saturation (SpO_2_) and heart rate (HR) were measured using a pulse oximeter (Nonin, model 2500) immediately before and immediately after the evaluation and intervention. Systolic blood pressure (SBP) and diastolic blood pressure (DBP) were measured on the upper limb using a stethoscope (Littmann, model Classic II S.E.) and a sphygmomanometer (BD, Curitiba, PR, Brazil). Respiratory rate (RR) was also recorded, and pain perception was assessed using the visual analog scale (VAS). All measurements and values obtained during the vital signs assessment were recorded on the data collection form.

After this evaluation, muscle strength was measured using dynamometry, and functional capacity was assessed with the 6‐min walk test (6MWT) at the beginning of physiotherapy treatment and at discharge. Both the initial assessment and the reassessment were preferably conducted by the same evaluator.

All physiotherapists responsible for treatment delivery were previously trained to standardize the intervention procedures. They were not involved in outcome reassessment, which was performed by blinded assessors.

### 2.4 Physiotherapeutic Assessment

Functional capacity, muscle strength, fatigue and quality of life were assessed.

#### 2.4.1 Six‐Minute Walk Test (6MWT)

Functional capacity was assessed using the 6‐min walk test (6MWT), which consists of walking at a moderate pace—without running—for 6 minutes, with the distance covered recorded in meters. Every 2 minutes, heart rate (HR), peripheral oxygen saturation (SpO_2_), and the Borg dyspnea scale were assessed, and encouraging phrases were provided, such as: “Very good!”, “You're doing very well”, and “We're almost there.” After 6 minutes, initial vital signs were rechecked, and the patient was monitored until returning to baseline parameters. The 6MWT was conducted according to standardized recommendations (American Thoracic Society 2002).

#### 2.4.2 Manual Dynamometry

Isometric muscle strength was evaluated using a digital hand‐held dynamometer (Instrutherm).

For the assessment, finger flexion was standardized with the right upper limb positioned in 90‐degree elbow flexion while seated. Three assessments were performed, and the best performance was selected.

#### 2.4.3 Administration of the Pediatric Quality of Life Inventory (PedsQL)—Multidimensional Fatigue Scale

A validated scale for assessing fatigue in pediatric patients is composed of three domains: general fatigue, fatigue and sleep, and mental fatigue. Each domain includes six response options ranging from mild to severe fatigue.

The PedsQL Multidimensional Fatigue Scale is an instrument designed to measure fatigue in children and adolescents (aged 2–18 years). It is used for both patients with acute or chronic health conditions and healthy populations.

The calculation and interpretation of the score follow a specific logic that reverses the raw score to facilitate reading on a scale from 0 to 100. Higher scores indicate better health (less fatigue).

The scale consists of 18 items divided into three dimensions: General Fatigue, which determines the overall feeling of tiredness (6 items), Sleep/Rest Fatigue, which refers to tiredness related to the quality or quantity of sleep/rest (6 items), and Cognitive Fatigue, related to mental tiredness, difficulty concentrating, and attention (6 items).

There are no right or wrong answers, and the domains of the scale were rated using numbers from 0 to 4, which correspond to the following scores: 0 corresponds to never, 1 to almost never, 2 to sometimes, 3 to often, and 4 to almost always (Eiser et al. 2003).

The participants selected the response that best reflected their reality at the time of answering and indicated their chosen response to the evaluator, who was not allowed to encourage or influence the participant's choice.

The items are reverse scored and linearly transformed into a scale from 0 to 100, as follows: 0 = 100, 1 = 75, 2 = 50, 3 = 25, 4 = 0.

To calculate scores for dimensions, the following were used: If more than 50% of the items on the scale are missing, the scale scores should not be calculated; Average score = Sum of items over the number of items answered; Total score: Sum of all items over the number of items answered on all scales; If more than 50% of the items on the scale are missing, the scale scores should not be computed; If 50% or more of the items are completed: Imputing the average of the completed items in a scale.

#### 2.4.4 Administration of the PedsQL Quality of Life Questionnaire—Cancer Module

A validated scale for assessing the quality of life in pediatric patients, adapted for use in cancer patients, consists of several items addressing pain, nausea, sleep disturbances, treatment‐related anxiety, concerns about self‐image, and communication.

There are no right or wrong answers, and the domains of the scale were rated using numbers from 0 to 4, which correspond to the following scores: 0 corresponds to *never*, 1 to *almost never*, 2 to *sometimes*, 3 to *often*, and 4 to *almost always* (Scarpelli et al. 2008).

The participant selected the response that best reflected their current reality at the time of answering and indicated it to the evaluator, who was not allowed to encourage or influence the participant's choice.

The items are reverse scored and linearly transformed into a scale from 0 to 100, as follows: 0 = 100, 1 = 75, 2 = 50, 3 = 25, 4 = 0.

To calculate scores by dimensions, the following criteria were applied: If more than 50% of the items on the scale are missing, the scale scores should not be calculated; Average score = Sum of the items divided by the number of items answered; Total score = Sum of all items divided by the number of items answered across all scales; If more than 50% of the items on the scale are missing, the scale scores should not be computed; If 50% or more of the items are completed: Impute the mean of the completed items for the scale.

In both scales, participants read the questions together with the professionals and responded independently according to their own perceptions.

### 2.5 Exercise Protocol

The physiotherapy intervention consisted of daily supervised and individualized sessions performed from hospital admission to discharge. In both groups, sessions lasted approximately 25 minutes and were conducted according to each participant's clinical condition and daily tolerance.

To promote consistency in intervention delivery, all treating physiotherapists received prior training and followed the same predefined intervention framework. Standardized components included the exercise modalities, targeted muscle groups, approximate session duration, aerobic intensity target, resistance‐training repetition range, respiratory exercise techniques, physiological monitoring procedures, perceived‐exertion target, and criteria for modifying, postponing, or suspending an activity. Individualization was permitted within this framework and included adjustment of external load, number of repetitions within the prescribed range, exercise duration, and seated or standing position according to the participant's daily clinical condition and tolerance. Thus, the core content and safety criteria were standardized, whereas the dose delivered during each session could be adapted in response to clinical status.

The intervention group received a structured protocol composed of aerobic exercise, resistance exercises, conventional respiratory exercises, and ambulation guidance. The aerobic component was performed using a cycle ergometer for approximately 5 minutes, targeting 50%–60% of the age‐predicted maximum heart rate, calculated as HRmax = 220 − age. Resistance exercises were performed using dumbbells for the upper limbs and ankle weights for the lower limbs. Three sets of 8–10 submaximal repetitions were prescribed for each exercise, according to participant tolerance.

The resistance exercises targeted functional muscle groups of the upper and lower limbs. Upper‐limb exercises included shoulder flexion and abduction, elbow flexion and extension, and wrist flexion and extension, performed mainly in a seated position using dumbbells. Lower‐limb exercises included hip flexion, knee extension, ankle dorsiflexion, and plantarflexion, performed in seated or standing positions using ankle weights when tolerated.

Conventional respiratory exercises were also applied and included sustained maximal inspiration, timed inspiration associated with upper‐limb elevation without resistance, and diaphragmatic proprioception. Participants were also encouraged and instructed to ambulate with the assistance of their caregiver when clinically stable.

Exercise progression and adjustment were individualized on a daily basis within the predefined protocol parameters. External load, duration, number of repetitions within the prescribed range, and exercise position were adjusted according to vital signs, signs of respiratory distress, pain, fatigue, treatment‐related symptoms, and patient‐reported tolerance. The Borg rating of perceived exertion scale was used to monitor intensity with a target between levels 7 and 8. When clinical instability or poor tolerance was identified, the treating physiotherapist followed the predefined adaptation criteria by reducing the external load or intensity, shortening the session, decreasing the number of repetitions, changing the exercise position, or postponing the activity.

Sessions were suspended if the participant presented nausea, dizziness, severe hypotension, heart rate exceeding the maximum limit during intervals, or pain rated two points higher on the Visual Analog Scale compared with baseline during protocol execution. No session had to be suspended for any participant included in the study due to adverse events related to the intervention (Carniel et al. 2023).

The minimal active physiotherapy comparator group received conventional respiratory exercises, including sustained maximal inspiration, timed inspiration associated with upper‐limb elevation without resistance, and diaphragmatic proprioception. Participants in this group were also encouraged and instructed to ambulate with caregiver assistance when clinically stable and were reassessed at hospital discharge.

### 2.6 Follow‐Up Plan

If a participant presented clinical changes requiring transfer to the intensive care unit (ICU) or another healthcare service, they would be withdrawn from the study. Similarly, participants who reported pain with a Visual Analog Scale (VAS) score two points higher than the baseline would also be excluded but re‐evaluated after 6 hours to determine the possibility of continuing with the protocol. Participants with hematological alterations (pancytopenia) could remain in the study, provided they exhibited no significant clinical manifestations such as desaturation, tachycardia, respiratory distress, bleeding, or severe hypotension (mean arterial pressure [MAP] below 60 mmHg), and were cleared by the medical team.

If the participant developed a fever, they were to be re‐evaluated within 6 hours to verify the continuation of the protocol. In the case of a surgical procedure or invasive examinations, re‐evaluation was to occur after 12 hours to assess the continuation of the study.

### 2.7 Statistical Analysis

The results are presented as mean ± standard deviation for continuous variables and as absolute and relative frequencies for categorical variables. The significance level was set at 5% for all analyses. The normality of continuous variables was assessed using the Shapiro‐Wilk test. Baseline characteristics were compared between groups using independent‐sample *t*‐tests for continuous variables and Fisher's exact test for categorical variables.

The effects of the intervention were analyzed using two‐way repeated‐measures ANOVA, considering group (comparator × intervention), time (baseline × discharge), and the group × time interaction. The main inference regarding intervention effects was based on the group × time interaction. Post hoc comparisons were conducted using Tukey's honestly significant difference test when appropriate. Effect sizes were reported as partial eta squared (η^2^p).

The main analysis was conducted using complete cases, including only participants with both baseline and discharge assessments. No imputation of missing outcome data was performed, and an intention‐to‐treat analysis was not conducted because final outcome data were unavailable for participants who died, withdrew, or were transferred to another healthcare facility before discharge assessment.

A post hoc sensitivity power analysis was performed to estimate the minimum detectable between‐group effect size based on the final sample included in the trial. This analysis was intended to characterize the statistical sensitivity of the completed study and did not replace a priori sample size calculation. Considering 30 participants, 15 participants per group, a two‐sided alpha level of 0.05%, and 80% power, the study would be able to detect only large between‐group effects in pre–post changes, approximately equivalent to Cohen's *d* = 1.06.

Because a baseline imbalance between groups was observed regarding the reason for hospitalization, an adjusted sensitivity analysis was also performed. Because of the small sample size and the low frequency of some hospitalization categories, the reason for hospitalization was dichotomized as chemotherapy admission versus non‐chemotherapy admission. Changes from baseline to discharge were compared between groups using linear regression models adjusted for chemotherapy admission.

No global adjustment for multiple testing was applied across the different outcomes and questionnaire domains. Therefore, analyses of secondary outcomes, individual questionnaire domains, and physiological variables were considered exploratory.

All statistical analyses were performed using SPSS version 20.0 (Chicago, IL, USA).

## 3 Results

A total of 45 participants were assessed for eligibility; however, 12 were excluded: three for being below the age range established by the study, six due to clinical instability, one participant who refused to continue in the study, and two who were transferred to another healthcare facility.

Regarding intervention exposure, the structured exercise protocol was delivered during hospitalization on days when participants were considered clinically eligible and able to tolerate the activities. However, complete standardized session‐level records were not available for all participants. Therefore, the total number of planned and completed sessions per participant, the proportion of prescribed sessions completed, missed sessions and their reasons, achieved exercise intensity and cumulative dose, and protocol deviations could not be reliably summarized. No intervention‐related adverse events requiring permanent discontinuation of the protocol were recorded.

A total of 33 participants were randomized, with 17 allocated to the intervention group (IG) and 16 to the minimal active physiotherapy comparator group (CG). Among those in the IG, one withdrew from the study and one died. Among those in the CG, one died, resulting in a final sample of 15 patients in each group.

The mean age was 12.00 ± 3.46 years in the total sample, 13.06 ± 3.19 years in the comparator group, and 10.93 ± 3.49 years in the intervention group. There were no significant differences in age, weight, or height between the comparator and intervention groups. Of the participants, 18 (60%) were male.

Regarding diagnosis, the total sample consisted of 18 participants (60%) with acute lymphoblastic leukemia, including 8 participants (53.3%) in the CG and 10 participants (66.7%) in the IG, and 12 participants (40%) diagnosed with lymphoma, including 7 participants (46.7%) in the CG and 5 participants (33.3%) in the IG.

The reasons for hospitalization varied among procedures (catheter placement), chemotherapy, and pathology follow‐up (examinations, assessments, and management of other mild clinical alterations), with a total of 14 participants (46.7%) hospitalized for chemotherapy—three participants (20%) in the comparator group (CG) and 11 participants (73.3%) in the intervention group (IG).

Only five participants (16.7%) received nutritional supplementation, and 16 participants (53.3%) underwent nutritional follow‐up. The mean hospital length of stay was 13.4 ± 10.1 days, indicating considerable between‐participant variability in the duration of hospitalization and, consequently, in the potential period of intervention exposure. The sample characteristics are detailed in Table 1.

**TABLE 1:** Sample characteristics.

Variable	Total (*n* = 30)	Comparator group (*n* = 15)	Intervention group (*n* = 15)	*p*‐value
Demographic				
Age (years)	12 ± 3.46	13.06 ± 3.19	10.93 ± 3.49	0.092
Weight (kg)	50.39 ± 21.95	57.73 ± 23.94	43.04 ± 17.58	0.066
Height (cm)	143.6 ± 21.24	144.13 ± 22.16	143.06 ± 21.04	0.893
Sex				
Male	18 (60%)	8 (53.3%)	10 (66.7%)	0.355
Diagnosis				
ALL	18 (60%)	8 (53.3%)	10 (66.7%)	0.355
Lymphoma	12 (40%)	7 (46.7%)	5 (33.3%)	0.355
Reason for hospitalization				
Procedure	2 (6.7%)	0 (0%)	2 (13.3%)	0.001
Chemotherapy	14 (46.7%)	3 (20%)	11 (73.3%)	0.001
Follow‐up	14 (46.7%)	12 (80%)	2 (13.3%)	0.001
Education level				
Incomplete elementary	15 (50%)	6 (40%)	9 (60%)	0.423
Completed elementary	5 (16.7%)	2 (13.3%)	3 (20%)	0.423
Incomplete high school	9 (30%)	6 (40%)	3 (20%)	0.423
Completed high school	1 (3.3%)	1 (6.7%)	0 (0%)	0.423
Supplementation				
Yes	5 (16.7%)	2 (13.3%)	3 (20%)	0.5
No	25 (83.3%)	13 (86.7%)	12 (80%)	—
Nutritional assessment				
Yes	16 (53.3%)	8 (53.3%)	8 (53.3%)	0.642
No	14 (46.7%)	7 (46.7%)	7 (46.7%)	—

Abbreviations: ALL, acute lymphoblastic leukemia; EL, elementary level; HS, high school; kg, kilogram.

Because a significant imbalance was observed between groups regarding the reason for hospitalization, an adjusted sensitivity analysis was performed. In the comparator group, 3/15 participants were admitted for chemotherapy and 12/15 for follow‐up, whereas in the intervention group, 11/15 participants were admitted for chemotherapy, 2/15 for follow‐up, and 2/15 for procedures. The distribution of hospitalization reasons differed significantly between groups (*p* = 0.001). When the reason for hospitalization was dichotomized as chemotherapy versus non‐chemotherapy admission, the imbalance remained significant (chemotherapy: 20.0% in the comparator group vs. 73.3% in the intervention group; Fisher's exact test, *p* = 0.009).

In the adjusted sensitivity analysis, changes from baseline to discharge were compared between groups using linear regression models adjusted for chemotherapy admission. The adjusted between‐group effect remained non‐significant for the main outcomes: total fatigue score (*β* = −6.62; 95% CI: −33.23 to 20.00; *p* = 0.614), total quality‐of‐life score (*β* = 13.99; 95% CI: −73.59 to 101.58; *p* = 0.746), 6‐min walk test distance (*β* = 2.37 m; 95% CI: −66.60 to 71.35; *p* = 0.944), and handgrip strength (*β* = 4.76 kgF; 95% CI: −2.12 to 11.64; *p* = 0.167). These findings indicate that, after adjustment for chemotherapy admission, the overall interpretation of the trial remained unchanged, with no clear evidence of superiority of the structured exercise protocol over the minimal active comparator for the main outcomes.

The effects of the intervention on fatigue and quality‐of‐life outcomes are summarized in Table 2. No significant group × time interactions were observed for the total scores or for any of the assessed fatigue and quality‐of‐life domains. Although temporal changes were observed for some outcomes, these changes did not significantly differ between groups. After recalculation according to the PedsQL scoring instructions, all PedsQL Cancer Module domains and total scores were within the expected 0–100 range.

**TABLE 2:** Effects of the intervention program on PedsQL indices—Multidimensional fatigue and quality of life scale (cancer module).

Variables	Comparator group			Intervention group			(F)		
							*p*		
							(ƞ^2^p)		
	Before	After	*p*	Before	After	*p*	Main effect: Time	Main effect: Group	Interaction: Time × Group
PedsQL—Multidimensional fatigue scale									
General fatigue	17.45 ± 5.27	54.18 ± 18.76	< 0.001*	17.75 ± 4.84	56.11 ± 18.95	< 0.001*	5.878	0.072	0.180
							0.022	0.791	0.675
							0.174	0.003	0.006
Sleep‐related fatigue	29.98 ± 8.50	61.11 ± 17.93	< 0.001*	31.14 ± 7.73	65.85 ± 18.58	< 0.001*	2.732	0.440	0.090
							< 0.001*	0.512	0.766
							0.879	0.015	0.003
Cognitive fatigue	38.63 ± 9.63	51.39 ± 14.58	< 0.001*	40.25 ± 10.26	53.06 ± 18.59	< 0.001*	5.046	0.133	0.053
							0.033	0.719	0.99
							0.153	0.005	0.002
Total fatigue score	55.55 ± 14.2	62.03 ± 13.20	0.51	58.3 ± 16.94	67.87 ± 14.99	0.029	5.939	0.204	0.057
							0.021	0.655	0.812
							0.175	0.007	0.002
PedsQL QOL—Quality of life scale (cancer module)									
Pain and hurt	68.33 ± 24.94	79.17 ± 26.16	< 0.001*	80.00 ± 16.90	83.33 ± 12.20	< 0.001*	0.948	0.635	1.334
							0.038	0.260	0.258
							0.932	0.022	0.045
Nausea	65.00 ± 18.52	73.33 ± 16.00	0.032*	68.00 ± 18.21	66.67 ± 16.97	0.484	0.697	0.146	1.334
							0.411	0.705	0.258
							0.024	0.045	0.045
Procedural anxiety	65.00 ± 21.40	89.64 ± 22.08	< 0.001*	52.78 ± 24.74	88.15 ± 19.38	< 0.001*	1.507	0.869	2.778
							0.230	0.359	0.107
							0.051	0.03	0.09
Treatment anxiety	70.55 ± 19.38	75.06 ± 19.67	0.158	64.45 ± 25.10	68.45 ± 18.56	0.208	0.015	1.453	0.114
							0.063	0.39	0.909
							0.118	0.027	0.004
Worry	52.78 ± 16.56	66.13 ± 16.99	< 0.001*	50.55 ± 23.03	61.15 ± 18.74	0.003*	0.013	0.304	0.356
							0.909	0.586	0.556
							0.007	0.011	0.013
Total quality of life score	64.33 ± 17.57	69.59 ± 16.20	0.212	63.15 ± 14.96	65.37 ± 15.67	0.594	1.063	0.550	0.260
							0.311	0.511	0.614
							0.037	0.016	0.009

Abbreviations: η^2^p, partial eta squared; F, F‐statistic; *p*, *p*‐value.

^*^ *p* < 0.05.

The effects of the intervention on functional capacity, handgrip strength, and physiological variables are summarized in Table 3. No significant group × time interactions were observed for six‐minute walk test distance or handgrip strength. Heart rate, oxygen saturation, and Borg scale values were considered physiological and perceived‐exertion responses rather than indicators of intervention effectiveness.

**TABLE 3:** Effects of the intervention program on physiological variables, dynamometry and the six‐minute walk test.

Variables	Comparator group			Intervention group			(F)		
							*p*		
							(ƞ^2^p)		
	Before	After	*p*	Before	After	*p*	Main effect: Time	Main effect: Group	Interaction: Time × group
6MWT distance (m)	168.47 ± 84.29	168.33 ± 84.62	0.995	254.67 ± 38.70	263.63 ± 33.26	0.654	0.1	0.455	0.106
							0.754	< 0.001*	0.747
							0.004	0.445	0.004
Handgrip strength (Kgf)	17.47 ± 8.62	17.67 ± 10.29	0.922	14.87 ± 8.26	17.53 ± 10.67	0.199	1.001	0.186	0.741
							0.326	0.669	0.397
							0.035	0.007	0.026
Initial HR (bpm)	96.33 ± 10.55	116.27 ± 12.79	< 0.001*	99.20 ± 8.50	121.27 ± 11.89	< 0.001*	0.066	1.666	0.163
							< 0.001*	0.207	0.69
							0.693	0.056	0.006
Initial SpO_2_ (%)	96.93 ± 1.28	96.00 ± 2.27	0.065	98.40 ± 0.83	98.20 ± 1.01	0.684	2.715	20.38	1.137
							0.111	< 0.001*	0.295
							0.088	0.421	0.039
Initial BORG	2.53 ± 1.19	4.73 ± 1.94	< 0.001*	1.33 ± 0.98	4.67 ± 2.50	< 0.001*	0.634	1.226	3.76
							< 0.001*	0.278	0.063
							0.762	0.042	0.118
Final HR (bpm)	96.93 ± 9.41	113.67 ± 10.29	< 0.001*	101.00 ± 9.30	116.60 ± 10.93	< 0.001*	0.769	1.428	0.067
							< 0.001*	0.242	0.797
							0.662	0.049	0.002
Final SpO_2_ (%)	96.00 ± 1.77	96.47 ± 2.07	0.383	97.27 ± 1.28	97.33 ± 1.68	0.9	0.512	4.438	0.288
							0.48	0.044*	0.596
							0.018	0.137	0.01
Final BORG	1.93 ± 1.03	3.87 ± 1.41	< 0.001*	1.20 ± 0.77	3.67 ± 1.80	< 0.001	1.021	1.235	1.337
							< 0.001*	0.276	0.257
							0.765	0.042	0.046

Abbreviations: F, F‐statistic; HR, heart rate; Kgf, kilogram‐force; m, meters; *p*, *p*‐value; SpO_2_, Oxygen saturation; η^2^p, partial eta squared; 6MWT, Six‐minute walk test.

^*^ *p* < 0.05.

## 4 Discussion

This randomized clinical trial evaluated whether a structured aerobic and resistance exercise protocol produced greater improvements than a minimal active physiotherapy comparator in hospitalized pediatric patients with oncohematological diseases. No significant group × time interactions were observed for fatigue, quality of life, handgrip strength, or functional capacity. Therefore, the study did not demonstrate superiority of the structured exercise protocol over respiratory exercises and ambulation guidance for the assessed outcomes. Although some outcomes changed over the hospitalization period, these temporal changes occurred without evidence of differential change between groups and cannot be attributed specifically to the additional aerobic and resistance components of the structured protocol.

Beyond statistical significance, the magnitude of the observed changes should be considered when evaluating their potential clinical relevance. For functional capacity, the mean six‐minute walk test distance changed from 168.47 to 168.33 m in the minimal active comparator group and from 254.67 to 263.63 m in the intervention group. These values correspond to changes of approximately −0.14 and +8.96 m, respectively, and an approximate descriptive difference in change of 9.10 m. However, the group × time interaction was not significant and its effect size was small (η^2^p = 0.004). In addition, a validated minimal clinically important difference for the six‐minute walk test has not been established for hospitalized pediatric oncohematological patients. Therefore, the observed change does not provide evidence of a clinically meaningful differential improvement attributable to the structured exercise protocol.

For handgrip strength, the mean change was approximately +0.20 kgf in the comparator group and +2.66 kgf in the intervention group, corresponding to an approximate descriptive difference in change of 2.46 kgf. Although this numerical difference may suggest possible preservation or improvement of muscle strength, the group × time interaction was not significant and the interaction effect size was small (η^2^p = 0.026). Furthermore, no directly applicable minimal clinically important difference for handgrip strength has been established in this population. Thus, the clinical importance of this change remains uncertain and cannot be attributed conclusively to the structured protocol.

Total fatigue scores increased by approximately 6.48 points in the comparator group and 9.57 points in the intervention group, with higher scores indicating less fatigue. The approximate descriptive difference in change was therefore 3.09 points, and the group × time interaction effect size was very small (η^2^p = 0.002). Although larger temporal changes were observed in some individual fatigue domains, these occurred in both groups and were not accompanied by significant differential effects. Consequently, the findings do not demonstrate a clinically meaningful benefit of fatigue specifically attributable to the additional aerobic and resistance exercises.

These non‐significant findings should be interpreted in the context of the limited statistical power. Because the final sample was sufficiently powered to detect only large between‐group effects, small or moderate differential effects may have remained undetected. Accordingly, the absence of significant group × time interactions indicates that superiority was not demonstrated but does not establish equivalence or rule out potentially relevant smaller effects.

For overall quality of life, scores increased by approximately 5.26 points in the comparator group and 2.22 points in the intervention group. The approximate difference in change was −3.04 points and therefore did not descriptively favor the structured intervention; the group × time interaction effect size was also small (η^2^p = 0.009). Some Cancer Module domains showed larger changes, particularly procedural anxiety, but the direction and magnitude of the changes varied across domains, and no significant group × time interactions were identified. Published minimal clinically important difference estimates for the generic PedsQL scales are not necessarily applicable to the PedsQL Cancer Module or the Multidimensional Fatigue Scale in hospitalized pediatric oncohematological patients. Accordingly, the observed temporal changes may have been perceptible to some participants but cannot be considered clinically meaningful effects of the structured intervention. Previous studies in pediatric and hematological oncology have reported heterogeneous effects of exercise interventions on fatigue, quality of life, muscle strength, and functional capacity. Some trials using longer interventions or follow‐up periods reported improvements in physical performance or fatigue‐related outcomes, whereas others found no significant between‐group differences. These inconsistencies may be explained by differences in intervention duration, exercise modality, clinical condition, comparator groups, and outcome measures. In the present study, the short hospitalization period and the use of a minimal active physiotherapy comparator may have reduced the ability to detect between‐group differences (Oechsle et al. 2014; Wehrle et al. 2019).

Previous studies have also reported mixed effects of exercise‐based interventions on muscle strength and functional capacity in oncohematological populations. Safran et al. (2022) found no between‐group difference in muscle strength when comparing resistance exercises combined with neuromuscular electrical stimulation with resistance exercises alone. In contrast, Duregon et al. (2019) reported improvements in functional capacity and muscle strength after a personalized exercise intervention delivered during hospitalization. These divergent findings reinforce that the effects of exercise may depend on intervention duration, supervision, exercise modality, clinical status, and the comparator condition.

It is also important to distinguish statistical significance from clinical relevance. In the present trial, statistical significance was assessed primarily through between‐group comparisons and group × time interactions. However, statistically significant within‐group changes do not necessarily indicate superiority of the structured exercise protocol or confirm clinically meaningful benefits. Conversely, the absence of statistical significance does not exclude the possibility of small or moderate effects, particularly given the limited sample size. Therefore, the clinical interpretation of the observed changes should be considered exploratory and should be supported in future studies by predefined clinically meaningful thresholds and larger adequately powered samples.

Jacobsen et al. (2014) evaluated exercise and stress management strategies during hematopoietic stem cell transplantation and reported improvements in physical and cognitive fatigue only among participants who received both interventions. However, the authors also highlighted that brief or self‐directed strategies may be insufficient during periods of high physical and emotional burden. In the present trial, the structured aerobic and resistance exercise protocol was delivered under close clinical supervision but did not demonstrate superiority over the minimal active physiotherapy comparator for the assessed outcomes.

Van Haren et al. (2018) implemented an intervention that included aerobic exercise using a stationary bicycle and resistance training targeting the back, abdomen, upper limbs, and lower limbs, whereas the control group performed no structured physical activity. A significant improvement in functional capacity was observed after 8 weeks. The authors also reported a significant improvement in total fatigue score in the intervention group compared with the control group (*p* = 0.022). Nevertheless, the findings of the present trial should be interpreted as exploratory because improvements occurred in both groups and the use of a minimal active physiotherapy comparator limited the ability to isolate the specific effects of the additional aerobic and resistance components. The use of a minimal active physiotherapy comparator may have contributed to improvements in both groups and reduced the contrast between groups, thereby limiting the ability to detect specific effects attributable to the additional aerobic and resistance components of the structured protocol.

The considerable variability in hospitalization duration likely resulted in heterogeneous cumulative intervention exposure. Participants with longer hospital stays potentially had more opportunities to complete exercise sessions, whereas those discharged earlier had a shorter exposure period. However, longer hospitalization may also have reflected greater clinical complexity, treatment‐related burden, complications, or poorer clinical status, which could independently impair fatigue, functional capacity, muscle strength, and exercise tolerance. Furthermore, because reassessment occurred at hospital discharge, the interval between baseline and follow‐up assessments differed among participants. These factors may have increased outcome variability and reduced the ability to detect differential changes between groups.

### 4.1 Primary Outcome: Quality of Life

Although some quality‐of‐life domains showed numerically relevant temporal changes, the absence of significant group × time interactions indicates that these changes cannot be attributed specifically to the structured exercise protocol. The total quality‐of‐life score improved numerically more in the comparator group than in the intervention group, and the domain‐level findings were inconsistent in direction and magnitude. Therefore, the present results do not provide evidence of a clinically meaningful differential benefit in quality of life.

In the present study, no significant between‐group difference was observed for the overall quality‐of‐life score, although favorable within‐group changes were noted in some domains. This finding is consistent with previous studies in hematological and pediatric oncology populations, in which exercise interventions did not consistently improve quality‐of‐life outcomes when compared with control or comparator conditions. Potiaumpai et al. (2021), for example, found no significant between‐group difference in quality of life assessed using the Functional Assessment of Cancer Therapy–Bone Marrow Transplant questionnaire. Similarly, Wehrle et al. (2019) found no between‐group differences in quality of life or nutritional status, although improvements in muscle strength were observed in the resistance training group. In contrast, Oechsle et al. (2014) reported improvements in quality of life and some aspects of muscle strength after exercise intervention. These divergent findings suggest that the effects of exercise on quality of life and physical outcomes may depend on factors such as intervention duration, exercise modality, clinical condition, treatment phase, comparator group, and outcome measure sensitivity.

Regarding muscle strength and physical function, previous studies have reported heterogeneous effects of exercise‐based interventions in pediatric and hematological oncology populations. Safran et al. (2022) and Duregon et al. (2019) reported improvements in muscle strength after structured interventions, while other studies demonstrated gains in functional capacity or mobility after resistance exercise, combined neuromuscular electrical stimulation, or supervised rehabilitation protocols. In the present study, however, no clear between‐group difference was observed for handgrip strength or functional capacity. This discrepancy may be explained by the short hospitalization period, limited exposure to the exercise protocol, small sample size, clinical heterogeneity, and the use of a minimal active comparator, which may have reduced the detectable contrast between groups.

Feasibility and safety are also important considerations in this population. Pahl et al. (2018) reported similar adherence between groups and no adverse events following the proposed activities, supporting the feasibility of exercise interventions during intensive treatment. In contrast, Jacobsen et al. (2014) found no measurable benefit from brief self‐directed exercise or stress management instructions during hematopoietic stem cell transplantation, suggesting that low‐intensity or insufficiently supervised strategies may be inadequate during periods of high physical and emotional burden. These findings are relevant to the present trial in which no intervention‐related adverse events requiring permanent protocol discontinuation were recorded among participants receiving the structured intervention. However, given the small sample size, this observation should not be interpreted as establishing the overall safety of the protocol or as excluding the possibility of uncommon adverse events.

### 4.2 Secondary Outcome: Functional Capacity Assessments and Results

The absence of significant between‐group differences in functional capacity in the present study is consistent with the heterogeneous findings reported in previous exercise trials involving pediatric or hematological oncology populations. Potiaumpai et al. (2021) also found no significant differences between the comparator and intervention groups in functional tests, although the intervention group showed improvement in aerobic capacity during follow‐up. Similarly, studies assessing the 6‐min walk test have suggested that functional performance may be influenced not only by exercise exposure but also by clinical factors such as treatment phase, hospitalization‐related deconditioning, and cardiovascular status. In our study, the considerable variability in hospitalization duration likely resulted in heterogeneous cumulative intervention exposure. Participants with longer hospital stays potentially had more opportunities to complete exercise sessions, whereas those discharged earlier had a shorter exposure period. At the same time, longer hospitalization may have reflected greater clinical complexity, treatment‐related burden, or poorer clinical status, which could independently impair functional capacity and exercise tolerance. Together with the active comparator condition, these factors may have increased outcome variability and reduced the ability to detect between‐group differences in functional capacity. Nevertheless, these findings support the need for individualized physiotherapy care, with exercise frequency and intensity adjusted daily according to clinical status, tolerance, and safety parameters.

The approximate 9.10‐m difference between the mean changes observed in the two groups was not accompanied by a significant group × time interaction and was associated with a small interaction effect size. In the absence of a validated clinically important threshold for hospitalized pediatric oncohematological patients, this difference should not be interpreted as a confirmed clinically meaningful improvement.

Hospital length of stay also varied considerably among participants, given that treatment sessions were scheduled daily from admission to discharge, hospitalization duration likely influenced the number of opportunities for intervention delivery and the cumulative exercise dose received. Participants with longer hospitalizations may have received greater intervention exposure; however, longer stays may also have been associated with greater disease severity, treatment‐related complications, clinical instability, or interruptions in exercise participation. In addition, discharge‐based reassessment resulted in variable intervals between baseline and follow‐up evaluations. Because complete session‐level data were unavailable, we could not quantify cumulative treatment exposure, evaluate dose–response relationships, or distinguish the effects of intervention duration from those of clinical status and hospitalization characteristics. This heterogeneity may have increased the variability of the outcome estimates and attenuated or obscured potential between‐group effects.

Previous studies evaluating physical performance and functional outcomes in pediatric or hematological oncology populations have reported mixed findings. Some trials observed preservation or improvement of physical performance after structured exercise interventions, whereas others found no significant between‐group differences in endurance, functional capacity, or physical function. Oechsle et al. (2014) reported improved physical performance in the intervention group and decline in the comparator group, while Wehrle et al. (2019) observed a smaller decline in the resistance and endurance groups compared with controls. In contrast, Jacobsen et al. (2014) and Van Haren et al. (2018) did not find significant differences in functional outcomes, although some favorable trends were observed in the intervention groups. In the present study, the intervention group showed a small increase in the 6MWT distance over time, but no significant between‐group difference was observed. Therefore, our findings should be interpreted as consistent with the heterogeneous literature, suggesting that measurable improvements in functional capacity may depend on intervention duration, training dose, supervision, clinical status, treatment phase, and outcome sensitivity.

### 4.3 Physiotherapy Implications and Future Research

From a physiotherapy perspective, the present findings indicate that the structured exercise protocol could be delivered under close clinical supervision in this small sample, with individual adjustments based on clinical status, tolerance, vital signs, and treatment‐related symptoms. No intervention‐related adverse events requiring permanent protocol discontinuation were recorded. Nevertheless, the limited sample size reduced the ability to identify uncommon adverse events, and the incomplete session‐level and intervention‐fidelity data restricted broader conclusions regarding safety and feasibility.

In many hospital settings, physiotherapy and exercise‐based interventions may be underused in this population because of concerns related to disease severity, treatment‐related symptoms, hematological instability, and clinical vulnerability. Although the structured aerobic and resistance exercise protocol did not demonstrate clear superiority over the minimal active comparator for most outcomes, no intervention‐related adverse events requiring permanent protocol discontinuation were recorded in this study. This finding should be interpreted descriptively and should not be considered as evidence of safety beyond the present sample. Nevertheless, feasibility should be interpreted cautiously because adherence, completed sessions, achieved exercise dose, and other measures of intervention fidelity were not systematically quantified. These findings reinforce the role of physiotherapists in preventing inactivity, encouraging mobility, monitoring physiological responses, identifying signs of intolerance, and adapting exercise prescription according to the patient's daily clinical condition.

Future studies should include larger and multicentre samples, longer intervention periods, and follow‐up after discharge to determine whether sustained exercise exposure produces measurable benefits in fatigue, quality of life, muscle strength, and functional capacity. Trials should also consider comparator groups that clearly distinguish usual care, minimal physiotherapy, and structured exercise interventions, as well as systematically report adherence, exercise dose, achieved intensity, missed sessions, adverse events, and clinically meaningful thresholds for the main outcomes. Future trials should also record hospital length of stay, the number of days on which participants were clinically eligible for exercise, planned and completed sessions, reasons for missed sessions, and cumulative exercise dose. When feasible, studies should use a standardized intervention period or analytically account for differences in exposure duration and hospitalization characteristics.

### 4.4 Limitations of the Study

Trials involving pediatric oncohematological populations are frequently constrained by recruitment difficulties and ethical considerations, which may result in small sample sizes. This limitation was present in the current study, in which the final analysis included only 30 participants. No priori sample size calculation based on a prespecified primary outcome and clinically meaningful between‐group difference was available. The post hoc sensitivity power analysis performed using the final sample size was intended only to characterize the detectable effect size of the completed trial and should not be interpreted as a substitute for prospective sample size planning. With 15 participants per group, the study had sufficient power to detect only large between‐group differences in pre–post changes, approximately equivalent to Cohen's *d* = 1.06. Therefore, the non‐significant group × time interactions may reflect either the absence of a substantial differential effect or insufficient statistical power to identify small or moderate effects. The possibility of type II error cannot be excluded, and the negative findings should be interpreted as failure to demonstrate superiority rather than evidence of equivalence or definitive absence of an intervention effect.

The single‐centre design and clinical heterogeneity of the sample also limit the interpretation and generalizability of the findings. Participants varied in age, diagnosis, reason for hospitalization, treatment stage, and clinical condition during admission. These factors may independently influence fatigue, quality of life, exercise tolerance, muscle strength, functional capacity, and physiological responses. In addition, children younger than 8 years were not eligible, and the sample included only participants with acute lymphoblastic leukemia or lymphoma. The findings may therefore not be generalizable to younger children, patients with other oncohematological diagnoses, different treatment phases, or greater clinical severity. Participants and treating physiotherapists could not be blinded because of the nature of the intervention. Although outcome assessors were blinded to group allocation, awareness of treatment assignment may have influenced participant‐reported outcomes or intervention delivery.

Another important limitation is the baseline imbalance between groups regarding the reason for hospitalization despite randomization. The intervention group included a substantially higher proportion of participants admitted for chemotherapy, whereas the minimal active physiotherapy comparator group mainly included participants admitted for follow‐up. Because chemotherapy admission may be associated with differences in fatigue, quality of life, exercise tolerance, muscle strength, functional capacity, and physiological responses, this imbalance may have confounded the estimated intervention effects. Although the adjusted sensitivity analysis accounted for chemotherapy versus non‐chemotherapy admission, adjustment for this single binary variable cannot eliminate residual or unmeasured confounding arising from other clinical differences between groups. In particular, the analysis could not fully account for treatment stage, disease severity, hospitalization acuity and duration, concurrent treatment‐related symptoms, complications, functional status, or other characteristics of the clinical admission. Moreover, dichotomizing reason for hospitalization did not capture the full heterogeneity of the original hospitalization categories. The small sample size and the low frequency of some categories precluded reliable adjustment for multiple covariates. Therefore, residual confounding remains an important limitation, and the adjusted sensitivity analysis should be interpreted as supportive rather than definitive when considering the absence of significant between‐group effects.

The active comparator condition should also be considered when interpreting the findings. Because both groups received respiratory exercises and ambulation guidance, the trial evaluated the incremental effect of adding aerobic and resistance exercises rather than the effect of physiotherapy compared with usual care, no treatment, or an inactive control condition. The active components received by both groups may have reduced the contrast between conditions and limited the ability to identify effects specifically attributable to the additional aerobic and resistance components. Consequently, the findings should not be generalized to comparisons between structured exercise and no physiotherapy or an inactive comparator.

Hospital length of stay varied considerably among participants, with a mean duration of 13.4 ± 10.1 days. Because sessions were scheduled from admission to discharge, hospitalization duration likely influenced the number of opportunities for intervention delivery, the cumulative exercise dose, and the interval between baseline and discharge assessments. Participants with longer hospitalizations potentially had greater intervention exposure; however, longer stays may have also reflected greater clinical complexity, treatment‐related complications, or poorer clinical status. Complete session‐level information regarding planned and completed sessions, missed sessions and their reasons, achieved intensity, cumulative exercise dose, and protocol deviations was not available. Although prior training and a common intervention framework were used to promote consistency across treating physiotherapists, no formal assessment of inter‐therapist agreement, independent observation of treatment delivery, competency testing, or standardized fidelity checklist was performed. Consequently, actual intervention exposure, adherence, dose–response relationships, and consistency of protocol delivery could not be objectively quantified. These limitations also restrict the interpretation of feasibility based solely on the absence of recorded intervention‐related adverse events.

The statistical approach also has limitations. The main analysis was based on complete cases and included 30 of the 33 randomized participants. No intention‐to‐treat analysis or imputation of missing outcome data was performed because discharge assessments were unavailable for the two participants who died and the participant who withdrew. Complete‐case analysis may introduce attrition bias, particularly if missingness is related to prognosis, clinical deterioration, or ability to tolerate the intervention. In addition, numerous outcomes and questionnaire domains were analyzed without a global adjustment for multiple testing, increasing the probability of type I error. Although Tukey's honestly significant difference test was used for post hoc comparisons when appropriate, it did not control multiplicity across the different outcome measures and domains. Secondary, domain‐level, physiological, main‐effect, and within‐group findings should therefore be interpreted as exploratory and hypothesis‐generating, with primary emphasis placed on the group × time interactions.

Finally, interpretation of clinical relevance is limited by the absence of validated thresholds for clinically meaningful changes in hospitalized pediatric oncohematological patients. Although minimal clinically important difference estimates have been published for some generic PedsQL measures, these thresholds cannot be directly generalized to the PedsQL Cancer Module or Multidimensional Fatigue Scale used in the present study. Similarly, directly applicable thresholds for the six‐minute walk test and handgrip strength are not clearly established for this population. Moreover, the study was not powered to detect small or moderate clinically relevant between‐group effects. Therefore, the observed changes should be interpreted as exploratory and hypothesis‐generating rather than as confirmed clinically meaningful benefits.

## 5 Conclusion

The structured therapeutic exercise protocol could be delivered to hospitalized pediatric oncohematological patients under close clinical supervision, without recording intervention‐related adverse events requiring permanent discontinuation. However, no significant group × time interactions were observed for fatigue, quality of life, handgrip strength, or functional capacity. Therefore, the superiority of the structured aerobic and resistance exercise protocol over the minimal active physiotherapy comparator was not demonstrated. Moreover, the magnitude and clinical relevance of the observed changes remain uncertain because population‐specific thresholds for clinically meaningful change are unavailable and the estimated differential changes between groups were small. The findings should be interpreted cautiously because of the small sample size, baseline clinical imbalance, variable hospitalization and intervention exposure, and limited information regarding treatment fidelity. Larger multicentre trials with standardized exposure periods, systematic fidelity monitoring, predefined clinically meaningful thresholds, and adequate statistical power are required.

## Author Contributions

C.F.C. and B.C.D.S. contributed to study conception, participant recruitment, intervention delivery, data collection, and preparation of the initial manuscript draft. H.F.L. contributed to data organization, literature review, and manuscript drafting. J.Z‐.R. contributed to methodological supervision, interpretation of findings, and critical revision of the manuscript for important intellectual content. R.D.R. contributed to study conception and design, methodological and statistical supervision, data interpretation, manuscript drafting, and critical revision. All authors have read and approved the final version of the manuscript.

## Funding

The authors have nothing to report.

## Ethics Statement

This is a randomized clinical trial. The study was submitted to and approved by the Research Ethics Committee (# 5.439.594). The protocol was registered with the Brazilian Registry of Clinical Trials (#RBR‐8sxnfyd). The research was conducted in the pediatric ward of the Mário Covas State Hospital in Sao Paulo, Brazil.

## Conflicts of Interest

The authors declare no conflicts of interest.

## Data Availability

The datasets generated and/or analyzed during the current study are available from the corresponding author upon reasonable request.
